# Construction and Performance Evaluation of *Nicandra physalodes* (Linn.) Gaertn. Polysaccharide-Based Nanogel

**DOI:** 10.3390/polym15081933

**Published:** 2023-04-19

**Authors:** Fangyan Liu, Chen Shen, Xuelian Chen, Fei Gao, Yin Chen

**Affiliations:** College of Food and Pharmacy, Zhejiang Ocean University, Zhoushan 316000, China

**Keywords:** *Nicandra physalodes* (Linn.) Gaertn, polysaccharides, structure, nanogel

## Abstract

The nanogels made from these polysaccharides and their derivatives are often used to construct drug delivery systems owing to their biocompatible, biodegradable, non-toxic, water-soluble, and bioactive characteristics. In this work, a novel pectin with unique gelling properties was extracted from the seed of *Nicandra physalodes* (NPGP). The structural research indicated that NPGP was a low methoxyl pectin with a high content of galacturonic acid. NPGP-based nanogels (NGs) were accomplished employing the water in oil (W/O) nano-emulsion method. The cysteamine containing reduction-responsive bond and integrin-targeting RGD peptide were also grafted onto NPGP. The anti-tumor drug doxorubicin hydrochloride (DOX) was loaded during the formation of NGs, and the performance of DOX delivery was studied. The NGs were characterized by UV-vis, DLS, TEM, FT-IR, and XPS. The results showed that the prepared NGs were nanosized (167.6 ± 53.86 nm), had excellent encapsulation efficiency (91.61 ± 0.85%), and possessed a fine drug loading capacity (8.40 ± 0.16%). The drug release experiment showed that DOX@NPGP-SS-RGD had good redox-responsive performance. Furthermore, the results of cell experiments revealed good biocompatibility of prepared NGs, along with selective absorption by HCT-116 cells through integrin receptor-mediated endocytosis to play an anti-tumor effect. These studies indicated the potential application of NPGP-based NGs as targeted drug delivery systems.

## 1. Introduction

Nanogels are intercross-linked three-dimensional chain networks composed of poly-ionic or nonionic polymers, with a size ranging from tens of nanometers to sub-microns, and are characterized by a high water content [1]. The advantageous properties of nanogels such as controllable size, easy preparation, swell ability, biocompatibility, hydrophilicity, and stimuli-dependent delivery render them next-generation drug delivery systems having obvious superiority over conventional approaches [2]. Therefore, nanogels have been exploited for efficient encapsulation and delivery system [3]. 

Natural polysaccharides that contain various reactive groups including –OH, –NH_2_, and –COOH could be modified and derivatized to fabricate functional nanogels such as hyaluronic acid, chitosan, and sodium alginate [4]. The nanogels made from these polysaccharides and their derivatives are often used to construct drug delivery systems owing to their biocompatible, biodegradable, non-toxic, water-soluble, and bioactive characteristics [5,6,7,8,9]. These examples show the interest of polysaccharides in the preparation of nanogel drug delivery systems. More and more natural polysaccharide resources have been developed and utilized to prepare nanogel [10,11,12,13].

Pectin, a major component of plant cell walls, is a kind of anionic polysaccharide that is mainly composed of α-(1-4)-D-GalA units and can also be used as a drug delivery system [14]. Several studies showed that pectin-based carriers can prevent the release of drugs in the stomach but start release in the intestine. The utilization of pectin as pH-triggered and time-release colon-specific drug delivery systems are discussed [15]. In addition, due to good bioadhesive force, pectin-based hydrogel showed the potential to adhere to human tissue [16]. Thus, great gel property, biocompatibility, and mechanical strength make pectin a promising natural material for drug delivery systems. For instance, the presence of pectin in honokiol-encapsulated self-assembled nanoparticles ensured higher cytotoxicity than a pristine drug on a sialoglycoprotein receptor overexpressing HepG2 cells [17]. However, the use of an ideal nano drug delivery system is decided primarily based on the biophysical and biochemical properties of the material [18]. The physical and chemical properties of pectin from different sources are very different [19]. As a novel source of pectin, *Nicandra physalodes* (Linn.) Gaertn. (NPG) is an erect annual herb of the Solanaceae family and naturally originates from Peru, but it is also distributed in Yunnan, China [20]. The seeds of NPG contain a layer of abundant pectic polysaccharide within the episperm. After fully soaking, NPG polysaccharide (NPGP) can be readily dissolved in water by manually rubbing out the swollen episperm. The pectin in NPG can form self-supporting and ordered gels, especially in the presence of Ca^2+^ ions. When adding a small amount of calcium hydroxide and holding the NPGP solution at room temperature for 20–30 min, a homogeneous, soft, and elastic gel can be obtained. Thus, the high content, simple extraction process, single composition, and excellent physicochemical properties make pectin in NPG a promising application in the food and medical fields.

Nanotechnology and the green chemistry route is widely encouraged by offering multiple benefits in treating chronic human diseases by site-specific, target-oriented delivery systems and lessening the side effects [21]. In addition to the target specificity of polysaccharides, targeting tumor cells or vasculature can be further improved by embedding receptor-binding peptides such as L-arginine-L-glycine-L-aspartic acid (RGD), which specifically binds to overexpressed integrin αvβ3 in tumor-associated neovasculature [18]. Thus, in the present work, we selected RGD peptide as the targeting molecule for tumors and employed NPGP as a carrier to explore its potential as a functional natural material for the targeted delivery of DOX. In order to expand the application of pectin in a nano drug delivery system, the novel pectin NPGP, which had unique gelling properties, was chosen to prepare disulfide bonding crosslinked NGs. Their release profiles were characterized. Cellular uptake and antitumor activity of the delivery system were also evaluated using HCT116 cells (an integrin αvβ3 over-expressing cell line).

## 2. Materials and Methods

### 2.1. Materials and Reagents

*N. physalodes* were from Zhaotong City (Yunnan province, China). Doxorubicin hydrochloride (DOX), reduced glutathione (GSH), and cysteamine, were from Aladdin Industrial Co. (Shanghai, China). The 1-Ethyl-3-(3-dimethylaminopropyl) carbodiimide hydrochloride (EDC), N-hydroxysuccinimide (NHS), dithiothreitol (DTT), and 4,6-diamidino-2-phenylindole (DAPI) were from Solarbio Science & Technology Co. (Beijing, China). 

### 2.2. Extraction of Nicandra Physalodes Polysaccharide

The polysaccharide (NPGP) was extracted by water decoction and alcohol precipitation. In detail, the seeds of NPGP were pocketed and immersed in distilled water at 60 °C. After squeezing the swollen seeds, the obtained polysaccharide solution was concentrated and subsequently precipitated by adding ethanol (80%, *v*/*v*) at 4 °C overnight. Next, the precipitates were collected and redissolved in distilled water, followed by dialysis. Finally, the polysaccharide solution was lyophilized and stored.

### 2.3. Structural Characterizations of NPGP

#### 2.3.1. Chemical Compositions

The uronic acid content was determined by carbazole-H_2_SO_4_ method using GalUA as a standard, while the protein content was measured by the method of Folin-phenol, employing bovine albumin as the standard. The monosaccharide composition of NPGP was assessed by PMP pre-column derivatization HPLC analysis after complete hydrolysis by 2 M trifluoroacetic acid at 105 °C for 6 h [22]. Furthermore, the molecular weight and homogeneity of NPGP were evaluated through multiangle laser scattering-coupled HPGPC [23].

#### 2.3.2. FT-IR Spectrum Analysis

The characteristics of NPGP and its modifications were analyzed using an FT-IR spectrophotometer. Briefly, a mixture of samples (1 mg) and potassium bromide was ground and pressed into a pellet for FT-IR measurement from 4000 to 500 cm^−1^.

#### 2.3.3. Carboxyl-Reduction and Methylation Analysis

Considering the acidic characteristic of NPGP, methylation analysis was not convenient for directly obtaining useful linkage information. Therefore, a uronic acid reduction was performed prior to methylation analysis in accordance with the described method [24]. The effect of carboxyl-reduction was examined by monosaccharide analysis. Subsequently, the sample was methylated by Hakomori’s method, with appropriate modification. Afterward, the methylated NPGP was hydrolyzed, reduced, and acetylated. Finally, the acetate derivative was analyzed by GC-MS. The methylation procedure and analysis were repeated thrice to obtain accurate data.

#### 2.3.4. NMR Spectroscopy Analysis

NPGP was exchanged in D_2_O twice, dissolved in D_2_O with a final concentration of 50 mg/mL, and analyzed with an Agilent DD2-600 MHz NMR apparatus.

### 2.4. The Preparation and Characterization of Nanogel

#### 2.4.1. Synthesis of NPGP-Cysteamine-RGD (NPGP-SS-RGD) Conjugate

**Scheme 1:**
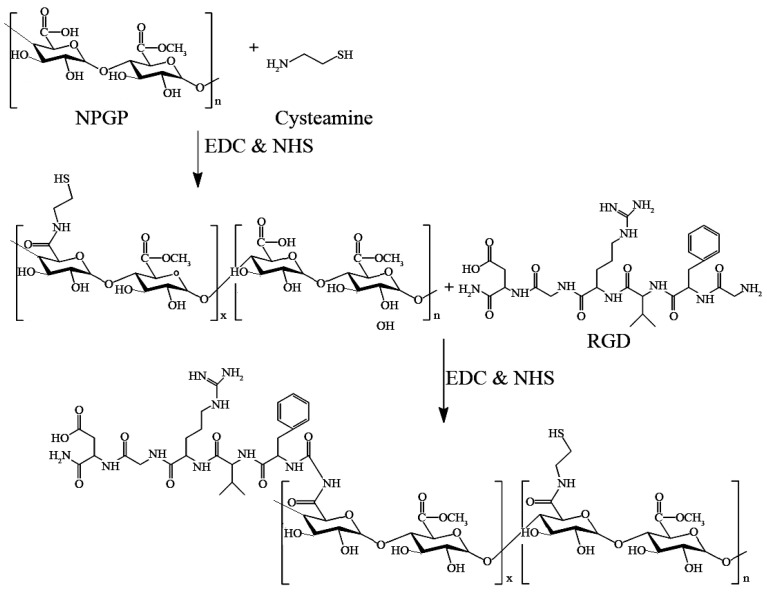
Synthesis route of NPGP-SS-RGD.

The covalent attachment of cysteamine and RGD peptide to NPGP was accomplished by an amidation reaction between the –NH_2_ of the cysteamine, RGD peptide, and –COOH groups of the polysaccharide (Scheme 1). Briefly, 128 mg of NPGP (1 mmoL) was hydrated in de-mineralized water and its carboxylic acid moieties were activated by the introduction of EDC (3 mmoL) and NHS (3 mmoL) for 2 h. The cysteamine was then added and stirred overnight in a molar ratio of 1:1 between the carboxylic acid group of NPGP and amino groups of the cysteamine. Next, an RGD peptide solution (1 mg/mL, 5 mL) was introduced and stirred for 24 h. The reaction system was further purified by removing unreacted reactants (MWCO = 3500) through dialysis in deionized water for 24 h. Then, the pH was raised to 7.4, contents were transferred to a conical flask, DTT was added, and the reaction was proceeded for 3 h. The as-obtained NPGP-SS-RGD conjugate was dialyzed against deionized water (pH 3.5) for an extended period. Eventually, the samples were lyophilized and stored at −20 °C. A similar method was adapted for the reaction of NPGP-cysteamine without RGD peptide [25].

#### 2.4.2. Preparation of Nanogels

NGs were fabricated employing the method of inverse microemulsion polymerization. Briefly, microemulsion solutions were prepared in 25 mL bottles by mixing a hexane stock solution (8 mL) of Span 80 (55.4 mg) and Tween 80 (55.4 mg) with an aqueous stock solution of NPGP-SS-RGD (15 mg) and water (1 mL). The emulsified solution was then stirred for 10 min with subsequent pulsing via Ultrasonic Homogenizer for 5 min in an ice bath, with the sonication cycle set to pulses of 5 s followed by a stop for 10 s. Following the addition of DOX·HCl, the solutions were pulsed for an additional 5 min, stirred for 24 h, and subsequently centrifuged at 8000 rpm for 3 min. The aqueous phase was obtained by repeatedly removing the supernatant and further extraction by acetone and hexane. Finally, the aqueous phase was centrifuged, and the precipitate was collected as an NG [26].

### 2.5. Characterization of NGs

The Ultraviolet-Visible (UV-vis) spectra of NPGP, cysteamine, RGD, and NPGP-SH-RGD were obtained using a UV-vis spectrophotometer. The FT-IR spectra of NPGP, NPGP-cysteamine, and NPGP-SS-RGD were measured. Meanwhile, the X-ray photoelectron spectroscopy (XPS) of NPGP, NPGP-cysteamine, and NPGP-SS-RGD was detected using a Thermo Scientific ESCALAB 250Xi XPS system that had a monochromatized AlKα (1486.7 eV) radiation source. The hydrodynamic diameter and surface charge of NGs were measured by a DLS instrument (Zeta sizer Nano-ZS90) at 25 °C, while their morphology was observed under a transmission electron microscope (TEM). The loading capacity was evaluated by deconstructing the structure of NGs through sonication in DMSO and subsequently measuring the content of DOX using UV-vis at 480 nm. Drug loading efficiency (DLE) and drug loading content (DLC) were calculated according to Equations (1) and (2), respectively.

DLE(%) = (weight of DOX)/(weigh of drug-loaded NGs) × 100%(1)

DLC(%) = (weight of DOX)/(weigh of DOX in feed) × 100%(2)

### 2.6. In Vitro Drug Release Studies

The drug release characteristics were examined in PBS (pH = 7.4) with different concentrations of GSH. The dialysis tubing (MWCO: 3.5–5 kDa), containing 2 mL of drug carrier solution (1.5 mg/mL), was placed into a glass beaker filled with 50 mL PBS. The glass beaker was kept in an incubated shaker at 37 °C with a vibrating speed of 100 rpm. At pre-determined intervals, aliquots of 3 mL of the external buffer solution were withdrawn and substituted with an equal volume of fresh PBS. The cumulative DOX release was determined by UV-vis at a wavelength of 480 nm.

### 2.7. Cell Viability Assay

HCT116 cells (human colon cancer cell line) were obtained from Zhejiang University as a gift. The cytotoxicity of blank NGs and DOX-loaded NGs in HCT116 cells was studied using MTT assay. In brief, HCT116 cells (8000 cells/well) were seeded in a 96-well plate and incubated for 48 h after the replacement of the cell culture media with fresh media containing the samples. Thereafter, 20 μL per well of MTT reagent (5 mg/mL) was added and incubated for 4 h. Subsequently, MTT-containing media were discarded, 150 μL of DMSO was added and shaken in dark for 10 min, and the absorbance was measured at 490 nm by a microplate reader. Cell viability was determined using the following equation:

Cell viability = (OD sample-OD blank)/(OD control-OD blank) × 100%

### 2.8. Cellular Uptake Assays

HCT116 cells (1.0 × 10^5^ cells/well) in 6-well plates were incubated for 4 h and 8 h with free DOX, DOX@NPGP-SS-RGD, and DOX@NGPG-SH NGs at an equivalent DOX concentration of 5.0 μg/mL. Then, the cells were subsequently washed thrice with PBS, fixed with 4% paraformaldehyde solution for a predetermined time, the cell nuclei was stained with DAPI (5 μg/mL) for 15 min, and washed again three times with PBS. Finally, the fluorescence images were captured using a laser scanning confocal microscope (LSCM).

### 2.9. Cellular Uptake Pathways of NGs

To evaluate the targeting ability of the RGD peptide, HCT116 cells expressing high αvβ3 levels were used. For quantitative analysis of cellular uptake, HCT116 cells were treated with a medium containing the inhibitors of endocytosis (free RGD peptide). After 2 h of incubation, the same concentrations of DOX-loaded NPGP-SS-RGD and NPGP-SH were added and further incubated for 4 h. Finally, the medium was expelled and the cells were rinsed three times with cold PBS to remove the un-uptaken nanogels. After that, a microplate reader was used to record the fluorescence intensity from a Dox-loaded nanogels group with excitation and emission wavelengths at 485 and 585 nm, respectively.

### 2.10. Statistical Analysis

SPSS 21.0 software were used for data analysis, and the data were expressed as mean ± SD. Statistical evaluation was carried by *t*-test and a value of *p* < 0.05 was adapted as statistically significant.

## 3. Results and Discussion

### 3.1. Composition and Structural Characteristics of NPGP

**Figure 1:**
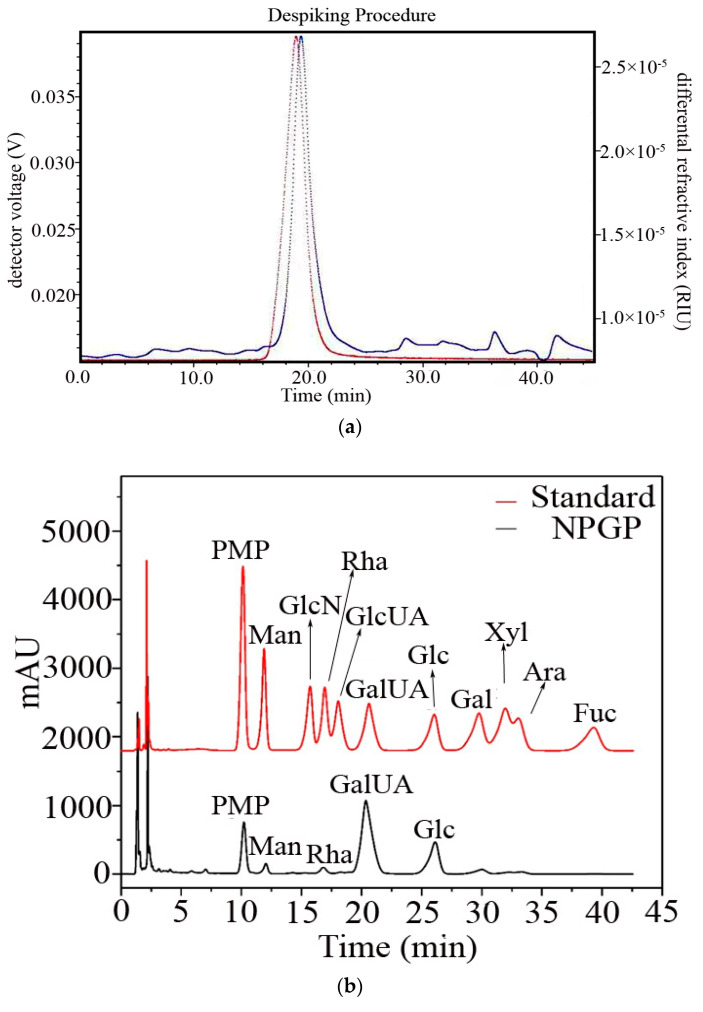

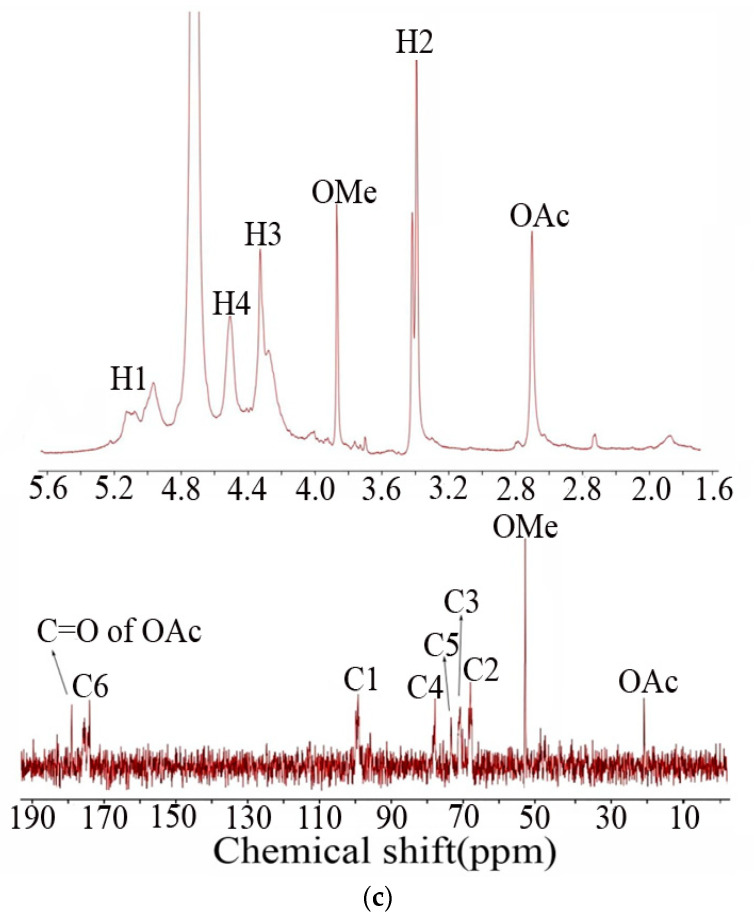
Monosaccharide composition (**a**) (The red line means the laser signal. The blue line means the refractive signal.), HPGPC chromatogram (**b**), and NMR spectra (**c**) of NPGP.

The water extraction and ethanol precipitation yielded 6.1% of NPGP. Chemical composition analysis presented 81.8% (*w*/*w*) uronic acid and 3.45% protein in NPGP. Further analysis showed that NPGP was composed of mannose (Man), rhamnose (Rha), galacturonic acid (GalUA), and glucose (Glc) at the ratio of 1.2:1:20.7:6.5 (Figure 1a), indicating that NPGP is an acidic heteropolysaccharide. The NPGP had comparable composition with that of pectin from the *Croton cajucara* leaf, which also contained Glc and Man in the side chains. However, the NPGP was almost devoid of Ara, Xyl, and Gal. The molecular weight (Mw) of NPGP was calculated as 1.28 × 10^6^ Da (Figure 1b), which was equivalent to the Mw of homogalacturonan (HG) [19].

Since the products of O-methylation alditol acetate derivatives of acid polysaccharides are difficult to volatilize in normal conditions of GC-MS, carboxyl-reduction of NGPG was afforded ahead of methylation analysis to acquire the substitution information of GalUA residues. The linkages of Gal could be utilized to deduce those of GalUA. As the Table 1, the linkages of →4)-Gal*p*-(1→ and →3, 4)-Gal*p*-(1→ indicated that GalUA residues were linked via O-4 and O-3, 4 and corresponded to 72% of the total residues. Through the ratio of →3, 4)-Gal*p*-(1→ and the total Gal linkages, we speculated that every 15 GalUA residues had a branch site at the O-3 position on average. The Glc residues had terminal and →4)-Glc*p*-(1→ linkages, while 2, 3, 6-Me_3_-Man suggested the presence of 4-linked Man*p* units. Methylation analysis revealed that NPGP was a slightly branched pectin-like polysaccharide with 1,4 linked GalUA as the main chain. The branch sites of the polysaccharide were at the O-3 position and occupied by 1, 4 linked Glc and Man.

The 1D (^1^H and ^13^C) NMR spectra of NPGP are presented in Figure 1c. The anomeric protons at 5.0 ppm in the ^1^H spectrum and anomeric carbon at 99.8 ppm in the ^13^C spectrum indicated the α configuration of the main residues of NPGP [27]. Based on the references, these major signals were ascribed to →4)-α-Gal*p*A-(1→. Furthermore, the typical signals at 3.85 ppm in the ^1^H spectrum and 52 ppm in the ^13^C spectrum were attributed to the H/C of methyl ester in NPGP. Based on the result of monosaccharide composition that no methylated sugar was found; the methylation position should be at O-6 position of GalUA. Further, the integration of the peak area in the ^1^H spectrum ascertained that 15% of the GalUA was methylated. The acetylated modification of NPGP was evident by the proton signals of acetyl at 2.75 ppm in the ^1^H spectrum and the carbon signal at 52 ppm in the ^13^C spectrum. The degree of acetylation was about 25%. Because of the sensitivity and resolution limitation of the NMR instrument and the relatively low proportion of other residues in NPGP, some signals failed to be reflected in NMR spectra. However, it was possible to determine the α configuration of Glc from the anomeric region of the ^13^C spectrum [28].

In conclusion, the results of structural analyses collectively verified that NPGP is a pectin-like polysaccharide mainly comprising an HG domain, which was constituted by →4)-α-Gal*p*A-(1→. In addition, a minor branched RG-II domain having complex side chains was identified in NPGP. The characterization also accentuated the difference in most of the glycosylation structure. Glucan and Mannan, exhibiting distinct degrees of polymerization, were linked to the O-3 position of the GalUA in the main chain of NPGP. The proportions of the methylated O-6 position of GalUA and acetylated polysaccharide were 15% and 25%, respectively.

### 3.2. Preparation and Characterization of NGs

**Figure 2:**
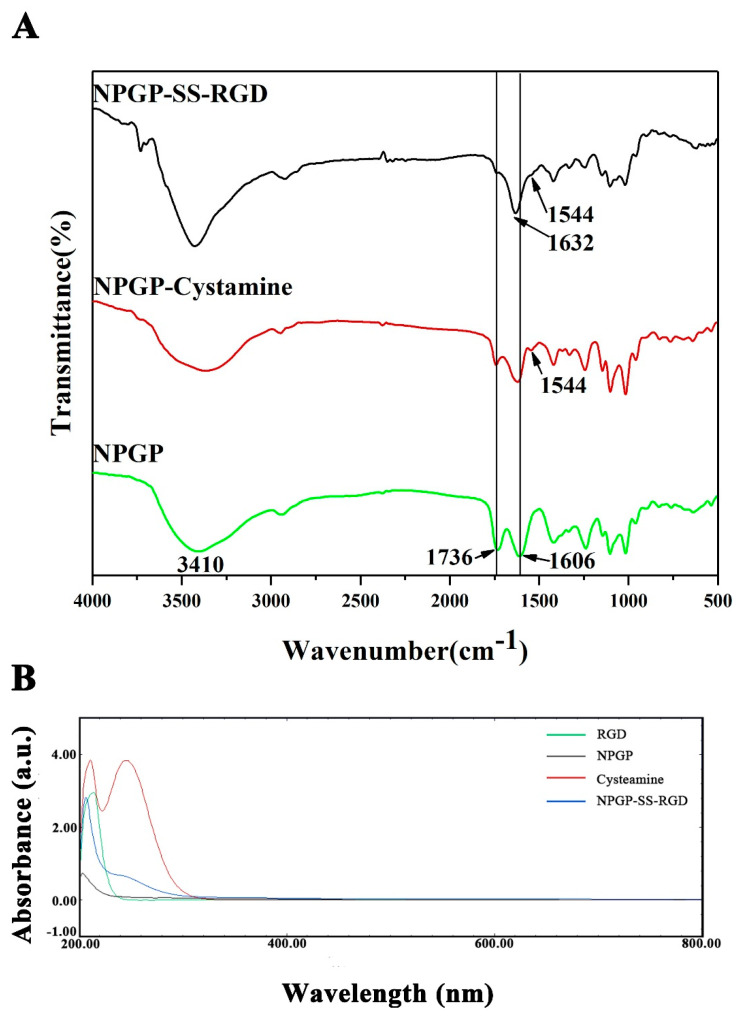

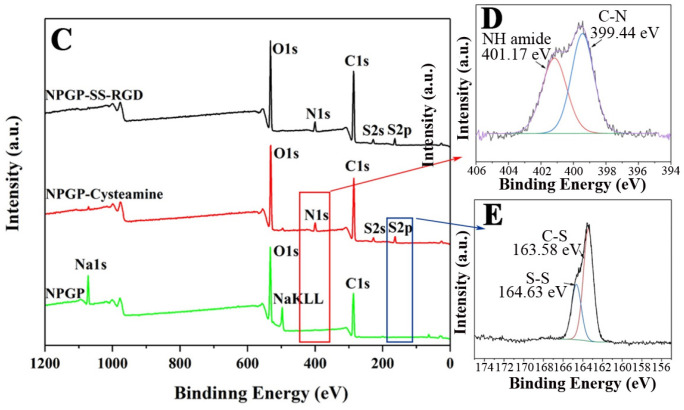
FT-IR spectra (**A**), UV-vis spectra (**B**), XPS survey spectra (**C**), N 1s spectrum (**D**), and S 2p spectrum of different formulations (**E**). The different color means different signals of the chemical bonds as labelled in the figures.

The chemical structures of NPGP, NPGP-cysteamine (NPGP-SH), and NPGP-SS-RGD were further characterized by FT-IR analysis. As shown in Figure 2A, the spectra of NPGP displayed the typical characteristic absorption peak of polysaccharides, with broad peaks around 3410 cm^−1^ (the hydroxyl stretching vibration). The peaks at 1736 cm^−1^ and 1606 cm^−1^ were attributed to the C=O stretching vibration of methylated and non-methylated carboxyl groups, respectively, which belonged to GalUA in NPGP. The reaction by cysteamine substantially diminished the peak at 1736 cm^−1^, shifted the peak to C=O stretching (amide II), and overlapped the peak at 1640 cm^−1^ (amide I). The spectrum of NPGP-SH-RGD showed a weakening of the absorption peak of 1746 cm^−1^ and the appearance of a typical amide peak at 1632 cm^−1^ (amide I), suggesting that the amino group on RGD peptide and the carboxyl group on NPGP were successfully linked together via amide bonds [29].

The conjugation was further validated with the help of UV-vis spectrums of NPGP, RGD, cysteamine, and NPGP-SS-RGD (Figure 2B). Cysteamine exhibited two absorption peaks at 207 and 225 nm, which could be ascribed to the n–σ* transition. Similar peaks in cysteamine-conjugated NPGP substantiated the cysteamine-mediated modification of NPGP. However, the binding of a small amount of RGD could not bring any noticeable change in the spectrums.

The elemental chemical status and chemical composition of the samples were analyzed using XPS to further confirm the functional groups on different formulations (Figure 2C). The peak for S 2p was detected at a binding energy of 164 eV (Figure 2E). These results implied the successful modification of NPGP-cysteamine or NPGP-SS-RGD surface using cysteamine. The chemical groups, along with bonding characteristics of NPGP-cysteamine, were further examined by high-resolution N 1s spectra. Figure 2D shows that the N 1s spectrum can be split into two peaks conferring energies at 401.17 and 399.44 eV, accredited to C–N and amidic NH, respectively. The XPS survey spectrum of the samples for C, O, N, and S elements (Table 2) exhibited an enhanced N 1s peak of NPGP-SS-RGD (Atomic ratio: 6.86%), signifying successfully connected RGD with NPGP-cysteamine [30,31,32].

**Figure 3:**
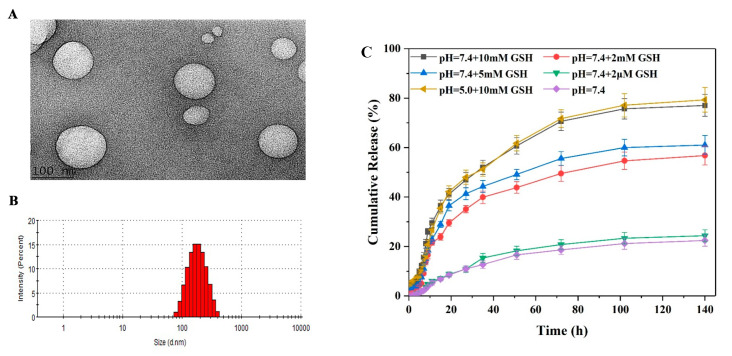
TEM image (**A**), size distribution profile (**B**) of NGs, and Cumulative release of DOX from DOX@NPGP-SS-RGD NGs (**C**).

The preparation of NGs was fabricated by the method of inverse microemulsion polymerization and the obtained NGs were studied by TEM and DLS analysis. The NGs demonstrated a spherical morphology, having an average particle size of about 100 nm (Figure 3A) with uniform size distribution (Figure 3B). The smaller particle size in TEM analysis than that in DLS evaluation could be ascribed to the shrinkage of the nanogel network resulting from the drying process of sample preparation.

### 3.3. Drug Loading and Drug Release Behaviors

The potential application of NGs as drug nanocarriers was evaluated by loading DOX into the NGs and subsequently determining their drug loading capacity and in vitro release profiles. The UV-vis absorbance of DOX at 480 nm fitted to a previously established calibration curve (Figure S1) displayed an outstanding DLE (91.61 ± 0.85%) and DLC (8.40 ± 0.16%) for NPGP-SS-RGD NGs. The DLE and DLC of NPGP-SH nanogel were 89.95 ± 0.42% and 7.88 ± 0.36%, respectively. The zeta potential of drug-free formulation increased from −36.8 ± 1.06 mV to −17.43 ± 0.50 mV for DOX@NPGP-SS-RGD NGs owing to the possible absorption of positively charged DOX on the surface of the NGs via electrostatic attraction [33]. Moreover, the loading of DOX increased the diameter and the size of drug-loaded nanogels (295.3 ± 70.98 nm) as compared with the blank nanogels.

The microenvironment of a tumor is characterized by high levels of glutathione (GSH) and low pH, especially in intracellular endosomes (pH 5.0–6.0) and lysosomes (pH 4.5–5.0), with reference to the normal cells [34]. Consequently, the disulfide links in NGs could be cleaved under intracellular reductive conditions to release their payload effectively with improved tumor-specificity. In this study, the release behaviors of DOX from DXO@NPGP-SS-RGD NGs were investigated at different GSH concentrations. As shown in Figure 3C, DOX presented multiple release behaviors at different GSH concentrations. The release rate increased with increasing GSH content, whereas a very slow DOX release was detected at physiological microenvironment (pH 7.4) or low GSH concentration (2 μM), conferring only approximately 20% of DOX release after 100 h. Interestingly, DOX was released rapidly at GSH strengths of 2 mM, 5 mM, and 10 mM, reaching a cumulative amount of 29.5%, 36.5%, and 41.1%, respectively, within 20 h. Moreover, in the same concentration of GSH at pH 7.4, the cumulative drug release was slightly higher than that in the nonreductive environment at pH 7.4. In addition to the sensitivity of DOX@NPGP-SS-RGD NGs towards the GSH stimuli, the results highlight the site-specific release without drug leakage during transportation through the physiologic environment [29].

### 3.4. In Vitro Cytotoxicity and Cellular Uptake

**Figure 4:**
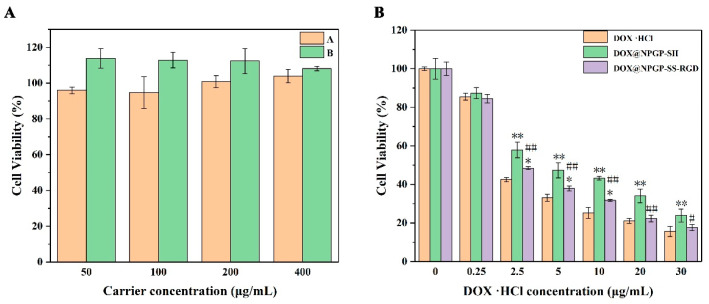
(**A**) Cell viabilities of HCT116 cells after 48 h incubation with NGs at different concentrations of NPGP-SH NGs (A) and NPGP-SS-RGD NGs (B). The data are expressed as mean ± SD, n = 3. (**B**) Anticancer activity of free DOX·HCl and DOX-loaded NGs against HCT116 cells as a function of DOX·HCl concentration after 48 h incubation. * *p* < 0.05; ** *p* < 0.01 versus DOX·HCl, # *p* < 0.05; ## *p* < 0.01 DOX@NPGP-SS-RGD versus DOX@NPGP-SH.

The in vitro cytotoxicity of blank nanogels, free DOX, DOX@NPGP-SS-RGD, and DOX@NPGP-SH NGs without RGD against HCT116 cells was determined by the MTT assay to investigate their biosafety and potential antitumor effects. It is noteworthy that equal concentrations of DOX-loaded and blank NGs were utilized to eliminate the effect of vehicles in the MTT assay. Figure 4A shows no obvious cytotoxicity in blank-treated cells for 48 h even at the highest concentration of 400 μg/mL, corroborating the good biosafety of synthesized NGs. Conversely, the viability of the cells exposed either to free DOX·HCl or DOX-loaded NGs was decreased with increasing drug concentration (Figure 4B). At a drug concentration of fewer than 20 μg/mL, the cytotoxicity of the pristine DOX·HCl was higher than all DOX-loaded NGs. However, when drug concentration was higher than or equal to 20 μg/mL, the DOX@NPGP-SS-RGD showed an anticancer effect equivalent to that of the free DOX·HCl and cell viabilities of less than 22.34%. DOX@NPGP-SS-RGD exerted higher toxicity to HCT116 cells as compared with DOX@NPGP-SH, possibly due to an integrin-assisted endocytosis mechanism. Although free DOX·HCl can effectively kill cancer cells, it has high toxicity to normal tissues/cells. These results suggested that RGD-mediated targeting increased the site-specific distribution and cytotoxicity of NGs in HCT116 cells [35,36].

### 3.5. Cellular Uptake of DOX-Loaded NGs

**Figure 5:**
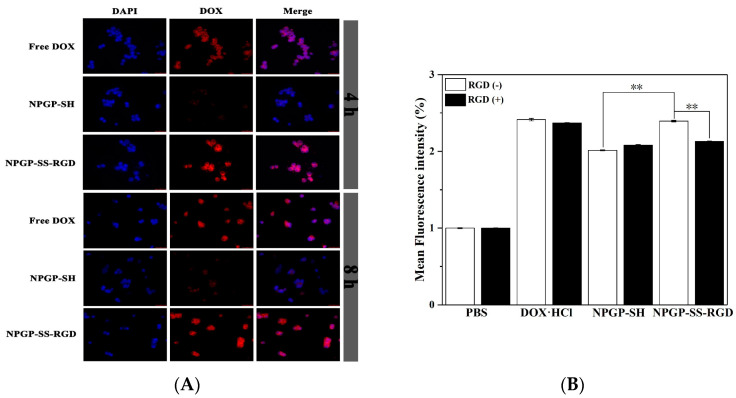
LSCM images showing intracellular uptake of DOX, DOX@NPGP-SH NGs, and DOX@NPGP-SS-RGD by HCT116 cells (**A**); cells were exposed to the indicated concentrations of DOX for 4 h and the levels of the intracellular DOX were analyzed by fluorescence intensity (**B**). ** *p* < 0.01 versus compared group.

The uptake and intracellular release of free DOX⋅HCl and DOX-loaded NGs in HCT116 cells were studied using LSCM. The cells were incubated separately with free DOX⋅HCl, DOX@NPGP-SH, and DOX@NPGP-SS-RGD for 4 or 8 h. The cell nuclei showed blue fluorescence after staining with DAPI, while DOX displayed red fluorescence. Strong red fluorescence in the pristine drug-treated cells indicated an effectively uptaken and fleetly internalized DOX (Figure 5A), which could be ascribed to its well-known permeation across cellular membranes and assimilation into the cell nuclei. Similarly strong red fluorescence in DOX@NPGP-SS-RGD-treated HCT116 cells affirmed effective uptake of NGs and, subsequently, successful offloading of the drug into the cell nuclei. Contrarily, the red fluorescence was very weak in HCT116 cells that were incubated with DOX@NPGP-SH NGs for 4 h and even for 8 h. This could reflect that the absence of RGD peptide limited the endocytosis of NGs. This result was also consistent with the results of cytotoxicity analysis.

### 3.6. Cellular Uptake Pathways of NGs

To further define the role of RGD peptides on targeting, HCT116 cells were incubated with free DOX, DOX@NPGP-SH, and DOX@NPGP-SS-RGD at DOX (5 μg/mL) in a culture medium comprising free RGD peptides. Figure 5B shows the mean fluorescence intensity of RGD-conjugated NGs compared with NGs in the absence and presence of free RGD peptides in the culture medium. The endocytosis in HCT116 cells against DOX@NPGP-SS-RGD was effectively inhibited by adding free RGD peptides, while, for the DOX@NPGP-SH NGs, no significant change was observed in the endocytosis of the cells that were incubated with a culture medium containing RGD peptide. Additionally, the endocytosis of HCT116 cells for DOX@NPGP-SS-RGD was significantly higher than that in DOX@NPGP-SH in RGD peptide-free culture medium. These results conformed with that acquired by LSCM. This competition analysis suggested that free RGD peptide could inhibit the cellular uptake of the DOX@NPGP-SS-RGD by competitive binding to the integrin on the cancer cell surface.

## 4. Conclusions

In this study, an acidic pectin-like heteropolysaccharide NPGP was extracted from *Nicandra physalodes* (Linn.) Gaertn. (NPG) seeds. NPGP was composed of Man, Rha, GalUA, and Glc at the ratio of 1.2:1:20.7:6.5 with a molecular weight of 1.28 × 10^6^ Da. The results of methylation GC-MS and NMR analysis showed that the main chain of NPGP was linked by →4)-α-D-Gal*p*A(1→. We employed a W/O nano-emulsion method to prepare NPGP-based NGs, which were further cross-linked with the cysteamine and RGD peptide via amide reaction. The NGs loading DOX were constructed with excellent physicochemical properties (small particle size, high encapsulation efficiency and DLC, desirable stability, and excellent biocompatibility). The drug release profile of DOX@NPGP-SS-RGD displayed a redox-responsive performance. The results of cell experiments showed that the prepared nanogel could be absorbed by HCT116 cells through integrin receptor-mediated endocytosis to exert targeted anti-tumor effects. Taken together, this evidence, the rationally constructed DOX loaded NPGP, could be a promising vehicle in cancer therapy. The pectin in *Nicandra physalodes* could be a good material to design natural polysaccharide-based nano drug carriers. The targeting and antitumor activity at the animal level require research and exploration in further study.

## Figures and Tables

**Table 1:** GC-MS data of partially O-methylated alditol acetates of NPGP.

Methylation Product	Linkage Type	Main MS (*m*/*z*)	Molar Ratio (%)
1,5-Ac_2_-2,3,4,6-Me_4_-D-Glc	Glc*p*-(→	101,117,129,145,161,205	5.9
1,4,5-Ac_3_-2,3,6-Me_3_-D-Man	→4)-Man*p*-(→	101,113,117,131,161,173,233	5.2
1,4,5-Ac_3_-2,3,6-Me_3_-D-Glc	→4)-Glc*p*-(→	101,113,117,131,161,173,233	15.0
1,4,5-Ac_3_-2,3,6-Me_3_-D-Gal	→4)-Gal*p*-(→	101,113,117,131,161,173,233	68.4
1,3,4,5-Ac_4_-2,6-Me_2_-D-Gal	→3,4)-Gal*p*-(→	87,101,118,129,180,234,305	5.5

**Table 2:** Element content (%) of NPGP, NPGP-SH and NPGP-SH-RGD.

Element Content (%)	C	O	N	S
NPGP	61.23	38.77	0	0
NPGP-SH	60.19	31.12	5.85	2.83
NPGP-SS-RGD	60.25	30.29	6.86	2.6

## Data Availability

The data are unavailable due to privacy and ethical restrictions.

## Supplementary Materials

The following supporting information can be downloaded at: https://www.mdpi.com/article/10.3390/polym15081933/s1, Figure S1: The standard curve of DOX.
