# Supplementary material for: Construction and Performance Evaluation of Nicandra physalodes (Linn.) Gaertn. Polysaccharide-Based Nanogel
Source: Polymers (Basel). 2023 Apr 19;15(8):1933. doi: 10.3390/polym15081933 (PMC10143989; doi:10.3390/polym15081933)
Supplement: Supplementary file 1 [file polymers-15-01933-s001.zip › polymers-2242120-supplementary.pdf]

Supplementary data

Construction and Performance Evaluation of *Nicandra physalodes* (Linn.) Gaertn. Polysaccharide-Based Nanogel

Fangyan Liu †, Chen Shen †, Xuelian Chen, Fei Gao and Yin Chen *

College of Food and Pharmacy, Zhejiang Ocean University, Zhoushan 316000, China; fangyanliuliu@outlook.com (F.L.); fei18957239291@outlook.com (C.S.); zhang17364621814@outlook.com (X.C.); gaofei565513128@outlook.com (F.G.)

* Correspondence: chenyin@zjou.edu.cn

† These authors contributed equally to this work.

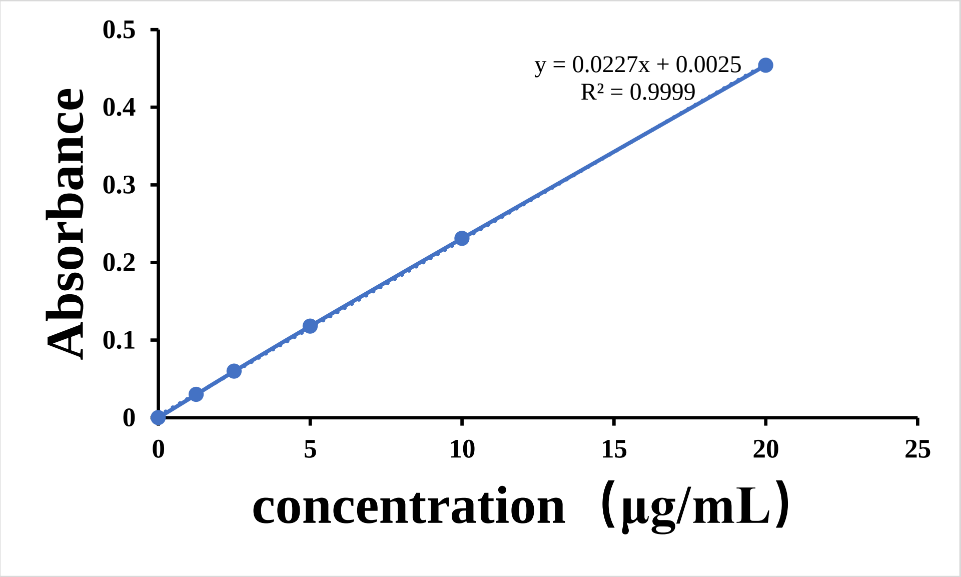

Figure S1. The standard curve of DOX.
